# A new approach to delineating clinical target volume for radiotherapy of glioblastoma: A phase II trial

**DOI:** 10.3389/fonc.2022.931436

**Published:** 2022-10-19

**Authors:** Yong Huang, Haixia Ding, Min Luo, Zhiqiang Li, Sirui Li, Conghua Xie, Yahua Zhong

**Affiliations:** ^1^ Department of Radiation and Medical Oncology, Zhongnan Hospital, Wuhan University, Wuhan, China; ^2^ Hubei Cancer Clinical Study Center, Wuhan University, Wuhan, China; ^3^ Department of Neurosurgery, Zhongnan Hospital, Wuhan University, Wuhan, China; ^4^ Department of Radiology, Zhongnan Hospital, Wuhan University, Wuhan, China

**Keywords:** radiotherapy, clinical target volume, glioblastoma multiforme (GBM), radiation volume, the white matter tracts

## Abstract

**Purpose:**

No consensus has currently been reached regarding the optimal radiation volume for radiotherapy of glioblastoma. Here, we have proposed a new delineation approach to delineating clinical target volume based on the relationship between the growth patterns of glioblastoma and neural pathways. Its safety and efficacy were evaluated in a phase II clinical trial.

**Methods:**

A total of 69 patients with histologically confirmed glioblastoma were enrolled. All patients underwent tumor resection, followed by focal radiotherapy and concomitant temozolomide (TMZ), and then received six cycles of adjuvant TMZ. The gross tumor volume (GTV) was defined as the surgical resection cavity plus any residual enhancing tumor, on contrast enhanced T1-weighted MRI. The clinical target volume (CTV) was delineated through our new approach.

**Results:**

The median recurrence-free survival (RFS) and overall survival (OS) were 11.4 months and 18.2 months, which were better than the previous reports. Relapse was found in 47 patients, of whom 41 patients (87.2%) failed in central, two patients (4.3%) failed in field, and four patients (8.5%) failed in distance. No marginal recurrence was found. Our regimen showed a trend of lower rates of marginal recurrence, and the brain volume of high-dose radiation fields in our regimen was similar to that of EORTC (p = 0.257).

**Conclusions:**

We have proposed a novel method for the delineation of clinical target volume by referencing the nerve fiber bundles for radiotherapy of glioblastoma. The results of the present phase II clinical trial suggest that this approach may be feasible and effective.

## Introduction

Glioblastoma (GBM) is the most common primary brain malignancy in adults (1). Maximal safe surgical resection followed by chemoradiotherapy is the standard treatment at present (2). However, the prognosis is still extremely poor. Local recurrence is the most common cause of failure (3).

Radiotherapy (RT) is one of the most important local treatments besides surgery, but currently, no consensus has been reached regarding the optimal radiation volume for high-grade gliomas (4). For instance, according to the recommendations of the European Organisation for Research and Treatment of Cancer (EORTC), the clinical target volume (CTV) was defined as the gross tumor volume (GTV) +2 cm (5). While in the guidelines of Radiation Therapy Oncology Group (RTOG), the initial field of CTV was defined as the peritumoral edema +2 cm and the boost field was defined as GTV +2.5 cm (6, 7). There are big differences between the two guidelines, no matter the radiation dose or target volume. However, several studies compared the differences in patterns of failure and survival between the two radiation techniques, and no significant difference was noticed (8–10). Larger RT fields do not show a survival advantage but lead to a higher risk of late neurological radiation-induced toxicity. However, according to the results of previous studies, smaller RT fields failed to treat all microscopic areas of infiltrating tumors (11).

The main growth characteristic of glioblastoma is infiltrative growth through the white matter tracts. Regions along the white matter tracts, especially those at the direction of the main fiber bundles, would have a higher risk of microscopic tumor cell dissemination (12). However, in current practice, the recommendation for the CTV definition is to add a 2 cm symmetrical margin to GTV or peritumoral edema in all directions, which hardly accounts for the growth characteristics of gliomas that are known from histopathological findings. Diffusion tensor imaging (DTI) has been reported to be able to detect white matter tracts and has been widely applied in surgical planning in glioblastoma patients (13–15).We have analyzed the relationship between the nerve fiber bundles and tumor growth patterns previously, and found that the application of the nerve fiber bundles will facilitate precise positioning of the radiotherapy target area (16). In this study, we traced the main white matter tracts by DTI and analyzed the growth patterns of GBM in relation to the main white matter tracts. Finally, we designed a detailed protocol for target delineation of the CTV and conducted a phase II clinical trial to evaluate the efficacy and safety of this strategy.

## Patients and methods

### Eligibility criteria

Approval of the study was obtained from the institutional review board of the Zhongnan Hospital, Wuhan University (Hubei, China). All patients signed informed consent forms before study entry. From August 2016 to December 2018, patients with glioblastoma were recruited for this study based on the following eligibility criteria: performance status of 0–1 (Eastern Cooperative Oncology Group performance status), histologically confirmed glioblastoma, no cerebrospinal fluid, and distant metastatic disease. All patients had adequate hematologic, hepatic, and renal function. Patients younger than 18 years old and older than 75 years; patients with a prior (i.e., within 5 years) or synchronous malignancy, other than non-melanoma skin cancer; and those with significant comorbidities were excluded.

### Study design and treatment

**Figure 1:**
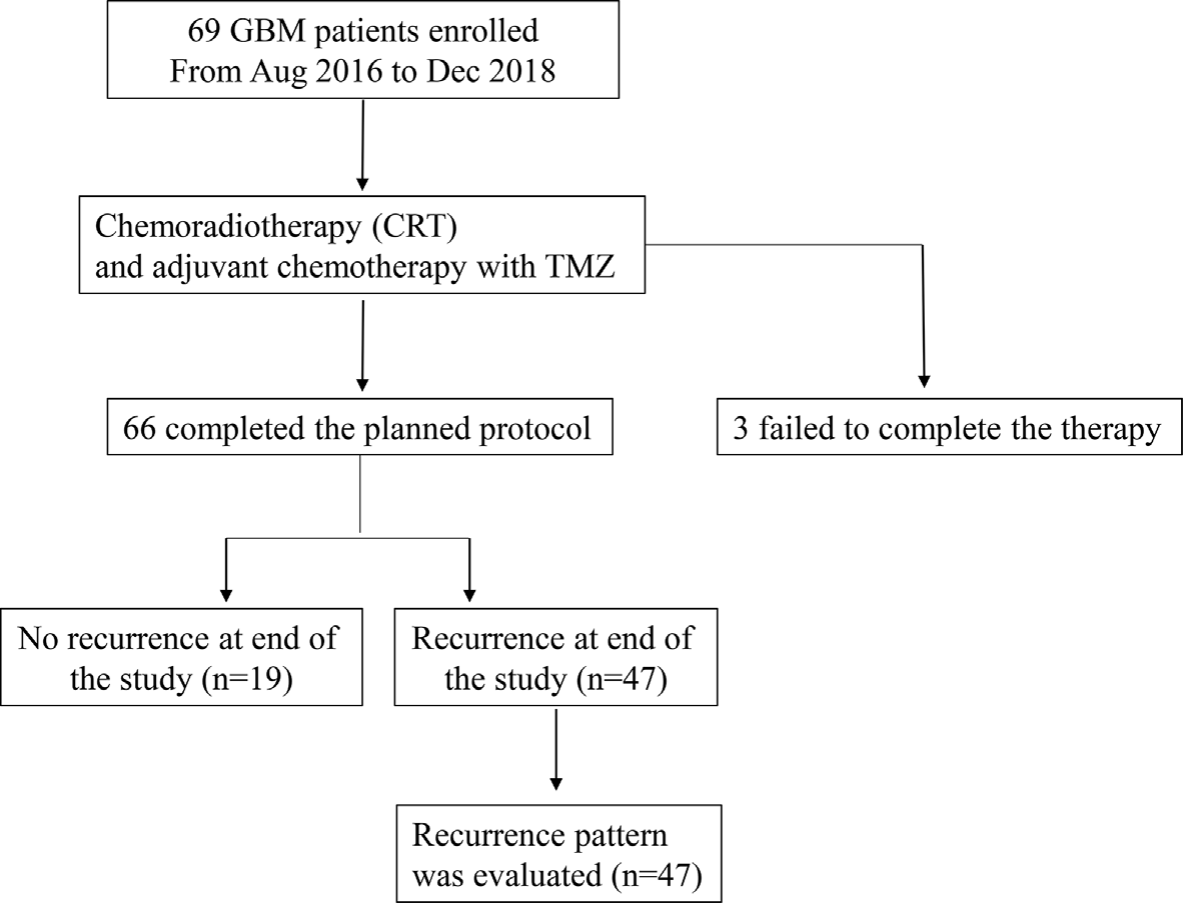
Flow chart of the study schema. GBM, glioblastoma.

Eligible patients received chemoradiotherapy (CRT) (PTV-GTV: 60 Gy at 2.0 Gy per fraction, five fractions per week for 6 weeks; PTV-CTV: 54 Gy at 1.8 Gy per fraction, five fractions per week for 6 weeks) with a Temozolomide (TMZ) regimen (75 mg/m^2^ per day during RT), followed by six additional cycles of TMZ (150 mg/m^2^ for the first cycle and 200 mg/m^2^ for the second to sixth cycles, on days 1–5, every 4 weeks). The flow chart of the study schema is shown in **Figure 1**.

### Target volume delineation and radiotherapy techniques

Patients were maintained in a supine position and immobilized with a thermoplastic elastomer film. All patients underwent a conventional and contrast-enhanced spiral CT scan (Siemens AG, Munich, Germany) with a 2-mm slice thickness from the vertex to the lower border of C3. Preoperative and postoperative (within 1 week and pre-radiotherapy) contrast enhanced MRI (contrast enhanced T1 + T2/FLAIR sequences) and DTI sequences were fused with the planning CT to aid target delineation. The target volume of GTV was defined as T1 enhancement and the surgical cavity without the inclusion of T2 abnormality. The target volume of CTV was delineated according to the methods described in the following paragraph. The PTV-GTV and the PTV-CTV included the GTV and CTV plus margins of 3 mm. Intensity-modulated radiotherapy (IMRT) techniques were delivered to the patients.

### Protocols of delineation for CTV

We established a detailed protocol for target delineation of the CTV based on brain anatomy, white matter fiber tract distribution, and the growth patterns of the tumor. Briefly, along the directions of the main nerve fiber bundles (**Table 1**), the CTV is defined as peritumoral edema plus 1 cm. While in other directions, the CTV is defined as GTV plus 2 cm and should be adjusted to anatomical borders such as the skull (0 mm, using bone window), ventricles (5 mm), falx (0 mm), tentorium cerebelli (0 mm), visual pathway/optic chiasm and brainstem (each 0 mm) and modified to include all regions of abnormal T2/FLAIR MRI signal. Deep brain white matter is the focus for RT target contour. Regions of normal uninvolved gray matter should be modified to be protected. **Supplementary Figure 1** provides an illustrative example of the new approach.

**Table 1:** List of main fiber tracts involved in different tumor locations.

Tumor location	Main fiber tracts involved
Frontal lobe	Superior longitudinal fasciculus Corticospinal tract The genu of the corpus callosum Corpus callosum body Inferior occipitofrontal fasciculus Unciform fasciculus Internal capsule
Temporal lobe	Arcuate fasciculus Inferior occipitofrontal fasciculus The splenium of the corpus callosumInferior longitudinal fasciculus Unciform fasciculus
Parietal lobe	Superior longitudinal fasciculus Corticospinal tract The splenium of the corpus callosum Corpus callosum body Internal capsule
Occipital lobe	Superior longitudinal fasciculus Inferior occipitofrontal fasciculus The splenium of the corpus callosumInferior longitudinal fasciculus Internal capsule

### Study endpoints

The primary endpoints were recurrence-free survival (RFS) and overall survival (OS). The secondary endpoint was pattern of relapse. RFS was calculated from the date of surgery to the date of the first locoregional recurrence. According to the DVH, if more than 95% of the recurrence volume was in the original high-dose field (isodose 95%—54 Gy), it was considered a central failure, whereas those within 80%–95% were designated as in-field, 20%–80% as marginal, and <20% as distant recurrences, respectively.

### Statistical analysis

Survival was investigated with the Kaplan–Meier method. All tests were two-sided, and significance was defined as a P-value less than or equal to 0.05. Statistical analyses were performed with the Statistical Package for Social Sciences, version 22.0 (IBM SPSS Statistics, Chicago, IL, USA).

## Results

Between August 2016 and December 2018, 69 GBM patients were enrolled. The median follow-up of patients included in the analysis was 15.2 months (range, 6.7 to 31.5 months). The baseline clinical characteristics of the patients are presented in **Table 2**. The median age was 52 years (range, 23–72 years), and 41 patients (59.5%) were male.

**Table 2:** Baseline characteristics of the patients.

Characteristics		No. of patients
Sex	Male	41
	Female	28
Age (years)	Median	52
	Range	23–72
KPS	Median	80
	Range	50–100
Location	Frontal	22
	Parietal	13
	Temporal	20
	Occipital	7
	Two lobes	7
Surgery	GTR	25
	STR	44
IDH status	Wild	63
	Mutant	6
MGMT status	Methylated	14
	Unmethylated	55

### DTI images

**Figure 2:**
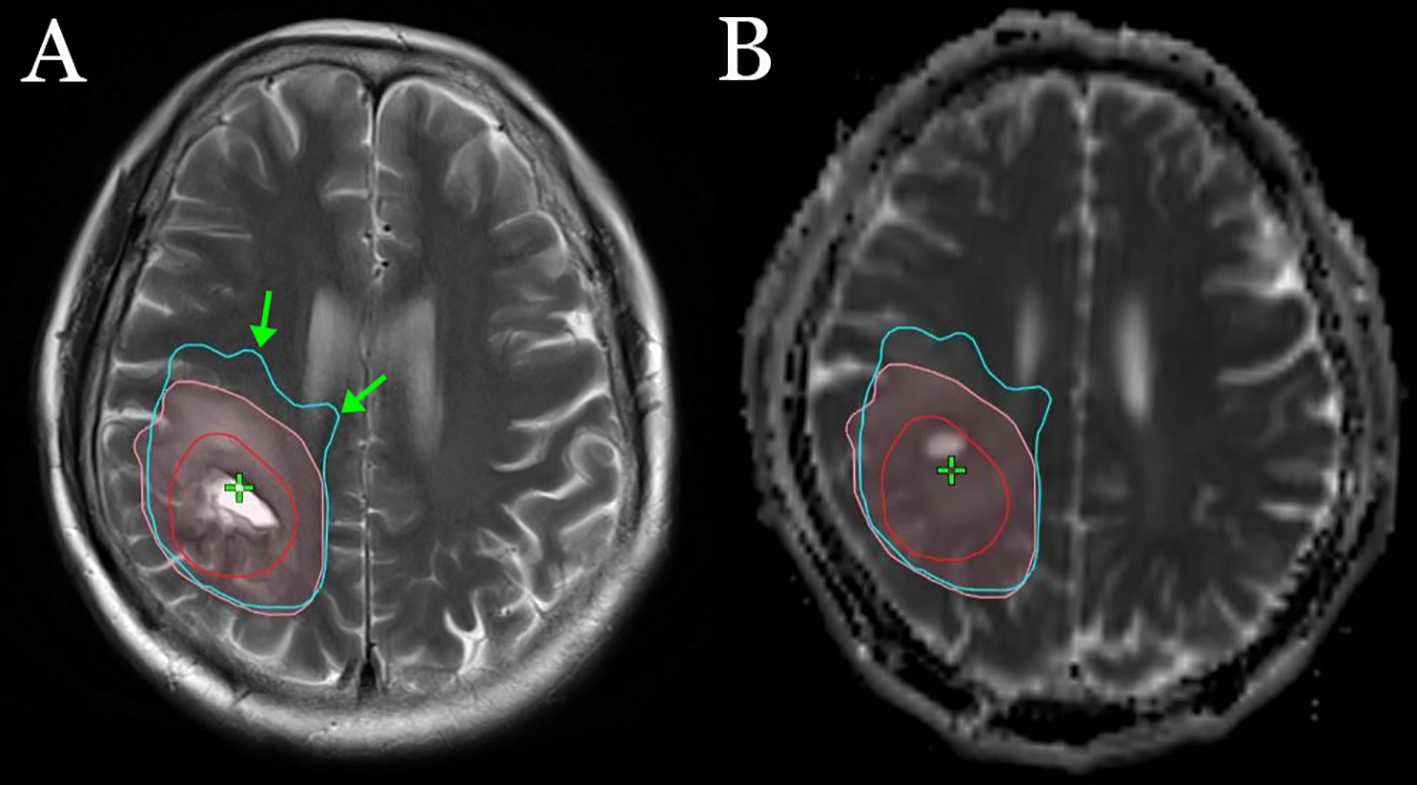
An example of the sites of tumor infiltration shown by T2 **(A)** and the DTI-p map **(B)**. The red line represents GTV, the pink lines represent the sites of tumor infiltration shown by T2, and the light blue lines represent the sites of tumor infiltration shown by the DTI-p map. The green arrow represents the depth of tumor infiltration shown by the DTI-p map was deeper than T2/FLAIR in the direction of adjacent main nerve fiber bundles.

The DTI-p (isotropic diffusion) map was reported to be an imaging correlate for the infiltrative edge of the tumor (17). A DTI-p map was taken in all 69 patients. The DTI-p map abnormality area was found to be inconsistent with T2/FLAIR in 60 patients (87%). The depth of tumor infiltration shown by the DTI-p map was found to be significantly deeper than T2/FLAIR in the direction of adjacent main nerve fiber bundles in 51 patients (73.9%) (**Figure 2**).

### Pattern of relapse

Sixty-six patients completed the planned protocol. Three patients failed to complete the therapy because of tumor recurrence during the period of adjuvant chemotherapy. During the follow-up period, relapse was found in 47 patients, of whom 41 patients (87.2%) failed in central, two patients (4.3%) failed in field, and four patients (8.5%) failed in distant recurrence. No marginal recurrence was found (**Table 3**). The MGMT promoter was methylated in 14 (36%) patients and unmethylated in 55 (64%) patients, respectively. Recurrence occurred central/in-field and outside in eight (80%) and two (20%) of patients with MGMT methylated status, and in 35 (94.6%) and 2 (5.4%) of patients with unmethylated status, respectively (P = 0.59).

**Table 3:** Comparison of pattern of relapse with different target delineation methods.

Recurrence pattern	Our plan No. of patients/total (%)	EORTC No. of patients/total (%)	P-value
Central	41/47 (87.2%)	79/105 (75.2%)	0.094
In-field	2/47 (4.3%)	6/105 (5.7%)	0.710
Marginal	0/47 (0)	6/105 (5.7%)	0.178
Distance	4/47 (8.5%)	14/105 (13.3%)	0.395

### Comparison of recurrence pattern

No significant difference was noticed between the recurrence pattern of EORTC (10) and our regimen. However, our regimen showed a trend of lower rates of marginal recurrence (**Table 3**). The comparison of brain volume exposed to radiation between our radiotherapy plan and the EORTC virtual plan is shown in **Table 4**. The brain volume of high-dose radiation fields in our regimen was similar to that of EORTC (p = 0.257).

**Table 4:** Comparison of brain volume of high-dose radiation fields between our radiotherapy and EORTC virtual plan.

	Our plan	EORTC virtual plan)	P-value
Total dose (Gy)	60	60	0.257
Median volume (CM3)	216.65	208.42	
Range	94.85–582.62	86.24–540.85	
Standard deviation (SD)	182.45	175.28	

### Survival

**Figure 3:**
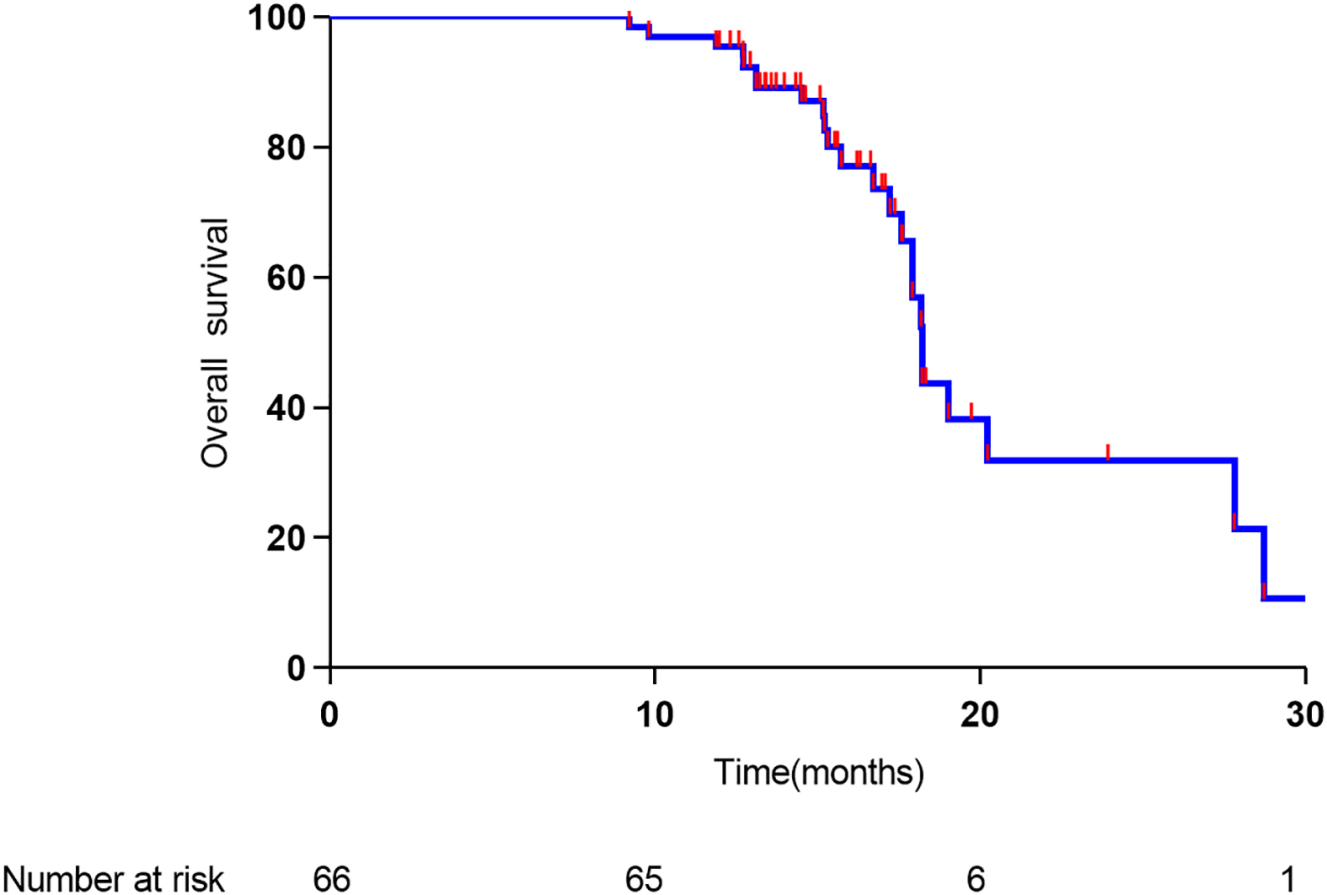
Overall survival of 66 study patients.

**Figure 4:**
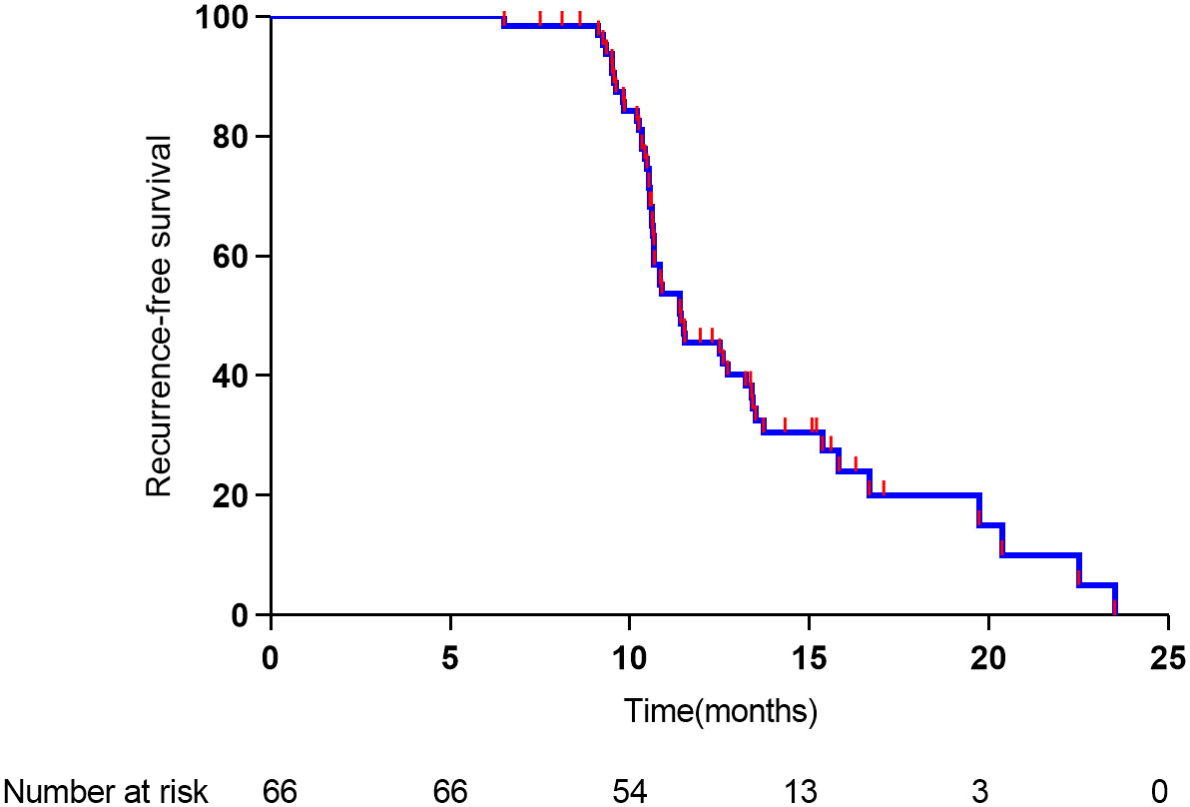
Recurrence-free survival of 66 study patients.

The median time to relapse was 11.4 months, and the median overall survival time was 18.2 months. As shown in **Figure 3**, the 1- and 2-year overall survival rates were 91% and 30%, respectively. As shown in **Figure 4**, the 1-year recurrence-free survival was 44.5%.

## Discussion

In the present study, we have established a new method for clinical target volume delineation by referencing the nerve fiber bundles in glioblastoma patients. Our phase II clinical trial of this approach showed a median RFS and OS of 11.4 months and 18.2 months, which were better than the previous reports (18, 19).

Among the 47 recurrence cases, 87.2% and 4.3% were relapsed in the central and in the field, respectively. No marginal recurrence was found and the distance recurrence was 8.5%. These preliminary results indicate that it may be feasible and effective to delineate CTV by using the white matter tracks for reference in glioblastoma patients.

**Figure 5:**
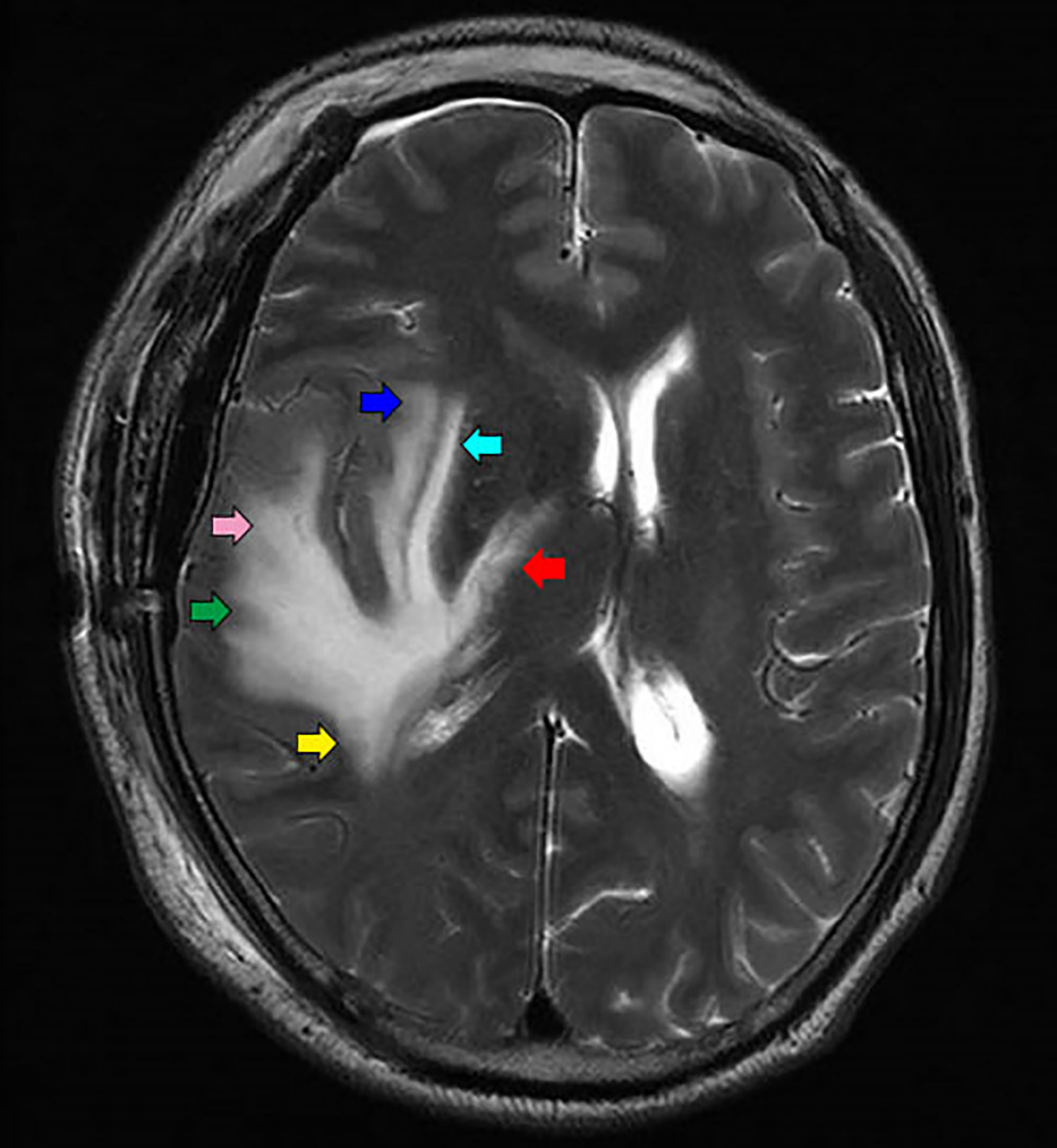
Different parts of edema is the representation of different nerve fiber bundles: Arcuate fiber (green arrow), inferior longitudinal fasciculus (pink arrow), external capsule (light blue arrow), Posterior limb of internal capsule (red arrow), tapetum of corpus callosum (yellow arrow), and extreme capsule (blue arrow).

It is well recognized that glioma cells can invade a distance away from the edge of a gross tumor by infiltration along white matter tracts (20). However, it would be very hard to accurately localize microscopic glioma infiltration by standard CT or MR imaging (21, 22). Price et al. examined the difference in tumor infiltration in different image-guided biopsy sites in 20 patients. In some cases, tumor infiltration was found in the MRI T2-weighted image normal region. They also analyzed the relationship between histologic examination and DTI and found that DTI was better than T2/Flair to direct local therapies for tumor infiltration (23). DTI displays the nerve fiber bundles by detecting the Brownian motion of water molecules (24). Tumor-induced changes in vascular permeability result in a large amount of water infiltrating into the interstitial space of the brain. These water molecules move along the nerve fiber bundle. Therefore, in most cases, the shape of the edema zone is exactly the same as the nerve fiber bundles shown by DTI. By analyzing the relationship between the shape of the edema and the nerve fiber bundles, we found that the nerve fiber bundle can be replaced by edema in some way (**Figure 5**). In addition, edema increases the gap between the fiber bundles, which is conducive to the spread of tumor cells. Therefore, on one hand, the edema zone provides the direction for the tumor to spread. On the other hand, the edema zone increases the interstitial space to facilitate the spread of the tumor, while the regions without edema are relatively difficult for tumor cells to infiltrate. Based on these points of view, the CTV in this study was recommended to include the edema.

As the cerebral falx and tentorium cerebelli are the continuation of the dura and play an important role as a barrier, it is rarely observed in clinical studies that tumors can directly infiltrate across them. Therefore, it is not recommended to include the normal brain tissue opposite the falx and tentorium cerebelli in the target volume. In this study, a 0 mm margin was applied to most of the falx and tentorium cerebelli barriers. However, on the plane of the corpus callosum body, tumor cells can spread from the corpus callosum to the opposite side, so 5 mm margin may not be enough. Margins in these regions should be adjusted according to the distribution of the nerve fiber bundles.

Most of the current target definitions for CTV are based on the analysis of the distance between the recurrence site and primary lesions. By analyzing CT data after whole-brain radiotherapy, Hochberg and Pruitt were the first to report that 80% of recurrences of GBM occurred within 2 cm of the primary tumor bed (25). These findings were also confirmed by later studies (26–28). Based on these results, isotropic margins were widely used in radiotherapy protocols (29). However, the infiltration of glioma is not isotropic but mainly along white matter tracks. Isotropic margins may increase the irradiation volume and lead to high doses of radiation to normal uninvolved brain tissue. Besides, according to the histopathological findings of previous studies (11, 12), tumor cells may spread further along main neural pathways than in other directions. Margins measuring 2 cm in these directions may not be enough to treat the possible microscopic areas of infiltrating tumor, which may lead to a high incidence of marginal and distant recurrence. Halperin et al. compared the extent of the neoplasm defined by histological and CT studies in 15 brains with GBM and found that a 3 cm margin around the edema was essential to cover the histologically identified tumor in all cases. Besides, the histological extent of the tumor was suggested to be non-isotropic but concentrated on main neural pathways (11). Jayamanne et al. reported on the correlation of first recurrence site and neural pathways in 100 recurrent temporal lobe GBM patients, many of the recurrences were reported to be predictable and more than half were involved with neural pathways (30).

**Figure 6:**
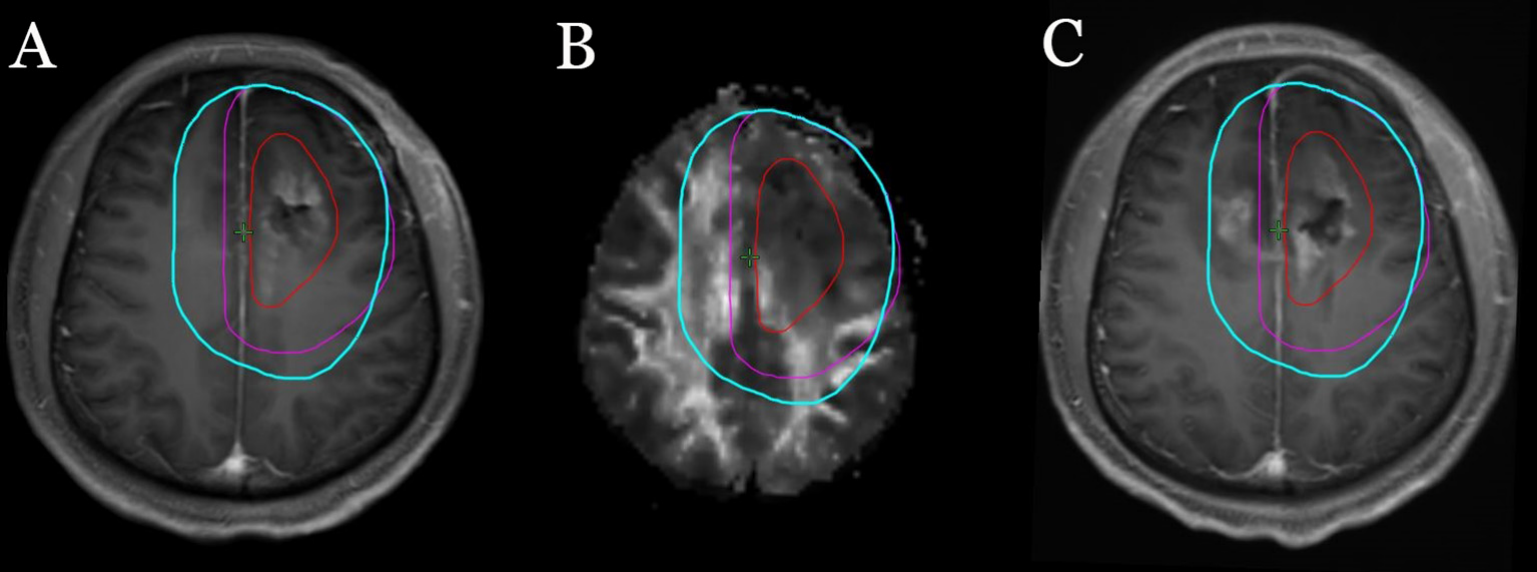
A patient with left frontal glioblastoma, the ipsilateral body of the corpus callosum was involved by tumor before surgery. The red line represents GTV, the purple lines represent CTV outlined according to the EORTC guidelines, and the light blue lines represent CTV outlined according to our methods. **(A)** Target area is shown in the postoperative T1 enhancement sequence. **(B)** Target area is shown in DTI Fa sequence. **(C)** The relationship between tumor recurrence and different target areas.

In this study, differentiated non-isotropic margins were adopted. At the directions of the main neural pathways, DTI can predict the sites of tumor progression more precisely than conventional MR images. We proposed that an additional 1 cm should be added to the CTV to treat all possible microscopic areas of infiltrating tumor. In other directions, a 2 cm margin around the GTV was added and modified to include all regions of abnormal T2/FLAIR MRI signal. The aim of this new approach was to try to make a balance between minimizing normal brain irradiation and treating infiltrating tumors as much as possible. Compared with RTOG guidelines, on the one hand, irradiated volume, especially the volume of normal brain, was reduced. On the other hand, the irradiated volume of the regions which are most likely to be infiltrated by tumors was ensured and the dose was increased. Compared with MD-Anderson’s recommendation, the irradiated volume along the main fiber bundles was increased. However, other directions, especially on the planes of the falx and tentorium cerebelli, were reduced. So the total volume of the CTV was similar. The results of this trial showed that nearly 92% of cases were relapsed in central and in the field, the distance recurrence was 8.5%, and no marginal recurrence was found. According to the analysis of the relationship between DTI and tumor recurrence pattern, we have found that in some patients the target area would be underestimated if DTI imaging was not used. In **Figure 6**, a patient with left frontal glioblastoma, the ipsilateral body of the corpus callosum was involved by tumor before surgery. According to the EORTC or RTOG guidelines, a 5 mm margin crossed over the cerebral falx to the opposite side would be the border of the CTV. However, according to the DTI imaging, the nerve fiber bundles of the bilateral corpus callosum are interconnected; therefore, the whole body of the corpus callosum should be included in the CTV. The analysis of the relationship between tumor recurrence and different target areas confirmed that DTI imaging was useful to guide the target delineation of radiotherapy. The increase of irradiated volume and dose to the high-risk area of microscopic glioma infiltration may be the main reason why marginal recurrence was reduced. All these results suggest that this approach may be feasible and safe.

The limitations of this study include the small size of samples, single-arm and non-randomized nature. More phase III trials should be carried out to validate our conclusion. Though DTI imaging technology can clearly show the main nerve fiber bundles, for tumors at a particular location, the number of adjacent nerve fiber bundles is large and they are intricate and cross-linked, so it is very difficult to predict which bundle or bundles of the nerve fibers will be the location or the distance of tumor spread. However, until we have sufficient imaging technology to predict where a recurrence is most likely to occur, the use of DTI is supported.

In conclusion, we have proposed a novel method for the delineation of clinical target volume by referencing the nerve fiber bundles for radiotherapy of glioblastoma. The feasibility and efficacy of this method have been demonstrated by the results of the present phase II clinical trial. When applying this strategy, practitioners should be very familiar with the distribution of the nerve fiber bundles.

## Data availability statement

The original contributions presented in the study are included in the article/**Supplementary Material**. Further inquiries can be directed to the corresponding author.

## Ethics statement

This study was reviewed and approved by the institutional review board of the Zhongnan Hospital, Wuhan University. The patients/participants provided their written informed consent to participate in this study. Written informed consent was obtained from the individual(s) for the publication of any potentially identifiable images or data included in this article.

## Author contributions

Analysis and interpretation: YH, HD, ML, SL, and YZ. Data analysis: YH, ZL, CX, and YZ. Experimental design: YH, HD, ML, and YZ. All authors contributed to the article and approved the submitted version.

## Funding

This work was partially supported by the National Natural Science Foundation of China (Grant No. 81641116).

## Conflict of interest

The authors declare that the research was conducted in the absence of any commercial or financial relationships that could be construed as a potential conflict of interest.

## Publisher’s note

All claims expressed in this article are solely those of the authors and do not necessarily represent those of their affiliated organizations, or those of the publisher, the editors and the reviewers. Any product that may be evaluated in this article, or claim that may be made by its manufacturer, is not guaranteed or endorsed by the publisher.

## Supplementary material

Supplementary Figure 1 The clinical target volume (CTV) delineation in a patient with left frontal lobe glioblastoma (subsequently delineated in T2, DTI-FA, CT/CE slices), GTV (red): T1 enhancement and surgical cavity without inclusion of T2 abnormality, CTV (light blue): peritumoral edema+1cm at main nerve fiber directions (yellow arrow **A**: Superior longitudinal fasciculus **B**: Corpus callosum body and Superior longitudinal fasciculus. **C**: splenium of the corpus callosum and Superior longitudinal fasciculus **D**: splenium of the corpus callosum, Inferior occipitofrontal fasciculus and Internal capsule **E**: Inferior occipitofrontal fasciculus **F**: unciform fasciculus), GTV+2cm at other directions, constrained at anatomical sites and modified to include all regions of abnormal T2/FLAIR MRI signal.
