# Supplementary figures and images for: A new approach to delineating clinical target volume for radiotherapy of glioblastoma: A phase II trial
Source: Front Oncol. 2022 Oct 19;12:931436. doi: 10.3389/fonc.2022.931436 (PMC9626993; doi:10.3389/fonc.2022.931436)

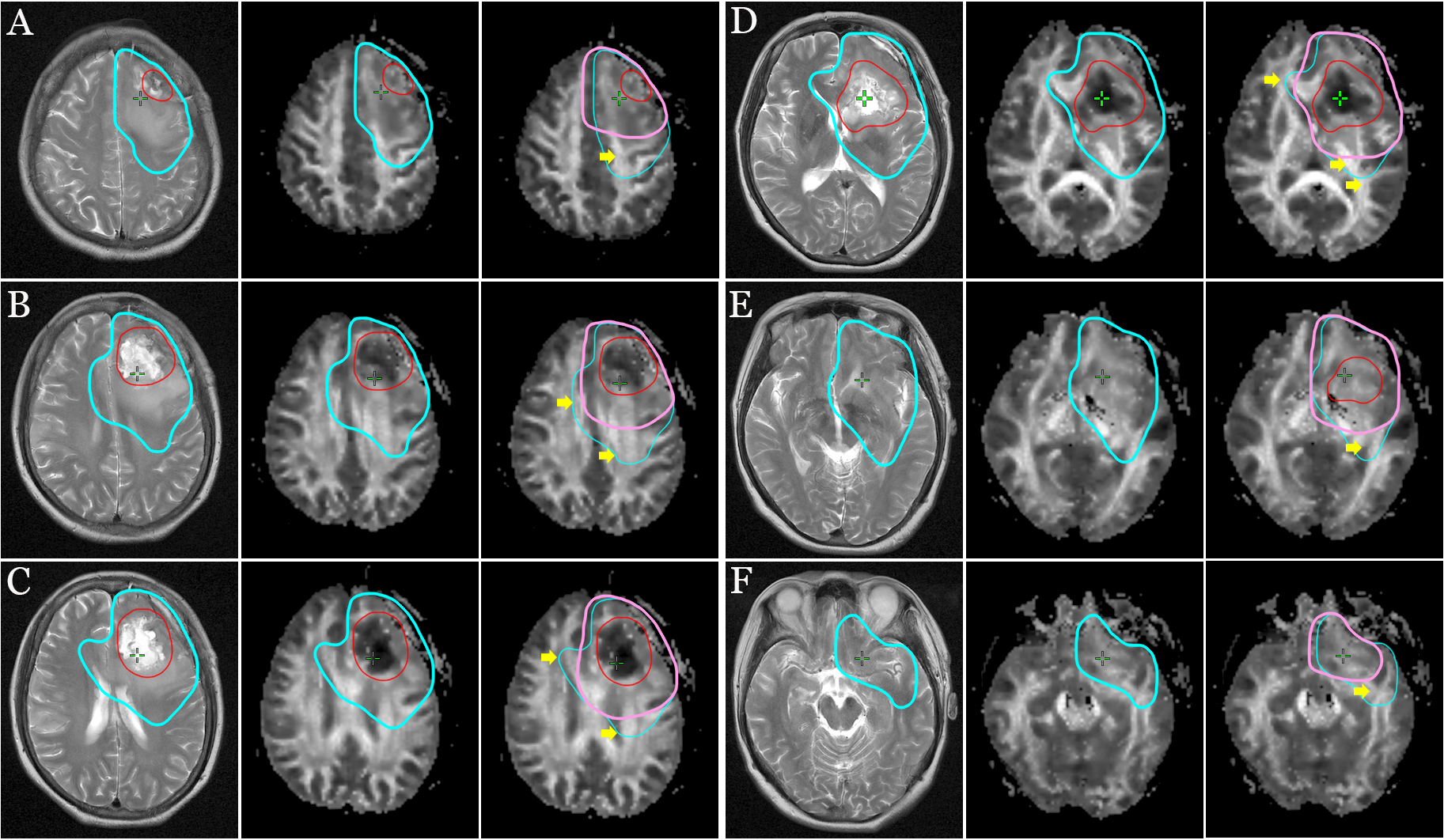

Supplement: Supplementary Figure 1 — The clinical target volume (CTV) delineation in a patient with left frontal lobe glioblastoma (subsequently delineated in T2, DTI-FA, CT/CE slices), GTV (red): T1 enhancement and surgical cavity without inclusion of T2 abnormality, CTV (light blue): peritumoral edema+1cm at main nerve fiber directions (yellow arrow A: Superior longitudinal fasciculus B: Corpus callosum body and Superior longitudinal fasciculus. C: splenium of the corpus callosum and Superior longitudinal fasciculus D: splenium of the corpus callosum, Inferior occipitofrontal fasciculus and Internal capsule E: Inferior occipitofrontal fasciculus F: unciform fasciculus), GTV+2cm at other directions, constrained at anatomical sites and modified to include all regions of abnormal T2/FLAIR MRI signal. [file Image_1.tif]
